# F-Actin Binding Regions on the Androgen Receptor and Huntingtin Increase Aggregation and Alter Aggregate Characteristics

**DOI:** 10.1371/journal.pone.0009053

**Published:** 2010-02-04

**Authors:** Suzanne Angeli, Jieya Shao, Marc I. Diamond

**Affiliations:** 1 Department of Neurology, University of California San Francisco, San Francisco, California, United States of America; 2 Department of Neurology, Washington University School of Medicine, St. Louis, Missouri, United States of America; Brigham and Women's Hospital/Harvard Medical School, United States of America

## Abstract

Protein aggregation is associated with neurodegeneration. Polyglutamine expansion diseases such as spinobulbar muscular atrophy and Huntington disease feature proteins that are destabilized by an expanded polyglutamine tract in their N-termini. It has previously been reported that intracellular aggregation of these target proteins, the androgen receptor (AR) and huntingtin (Htt), is modulated by actin-regulatory pathways. Sequences that flank the polyglutamine tract of AR and Htt might influence protein aggregation and toxicity through protein-protein interactions, but this has not been studied in detail. Here we have evaluated an N-terminal 127 amino acid fragment of AR and Htt exon 1. The first 50 amino acids of ARN127 and the first 14 amino acids of Htt exon 1 mediate binding to filamentous actin *in vitro*. Deletion of these actin-binding regions renders the polyglutamine-expanded forms of ARN127 and Htt exon 1 less aggregation-prone, and increases the SDS-solubility of aggregates that do form. These regions thus appear to alter the aggregation frequency and type of polyglutamine-induced aggregation. These findings highlight the importance of flanking sequences in determining the propensity of unstable proteins to misfold.

## Introduction

Spinobulbar muscular atrophy (SBMA) and Huntington disease (HD) are devastating neurodegenerative diseases. SBMA is caused by an expanded CAG trinucleotide repeat that encodes a long polyglutamine tract in the androgen receptor (AR) [1], while HD is caused by an enlarged polyglutamine tract in the huntingtin (Htt) protein [2]. Proteolytic cleavage of AR and Htt appears to generate toxic, N-terminal fragments [3], [4], [5], [6]. These are sufficient to recapitulate neurodegenerative phenotypes *in vivo* [7], [8]. N-terminal fragments of expanded AR and Htt readily aggregate *in vitro* and in cell-culture models, thus making them useful in biochemical studies [9], [10], [11]. While the aggregation and toxicity of polyglutamine proteins directly correlate with the length of the polyglutamine tract [11], flanking sequences are also clearly important [12], [13], [14], as are intracellular signaling pathways that act via protein interactions or post-translational modifications [10], [15], [16]. Emerging evidence from a variety of studies of aggregation-prone proteins associated with neurodegenerative diseases suggests that there is considerable diversity among aggregates that can be formed *in vitro* [17], [18], [19], and, moreover, that some protein aggregates are likely to be more toxic than others [19], [20]. Thus, protein interactions that alter aggregate conformation could play an important role in determining toxicity.

Indirect evidence implicates actin and/or actin-binding factors as an influence on polyglutamine-dependent aggregation of AR and Htt [10], [21], [22], [23], [24], [25], [26]. Y-27632, a rho-kinase (ROCK) inhibitor, reduces intracellular polyglutamine aggregation of Htt exon 1 and the N-terminal fragment of AR, termed ARN127 [10], [25]. Y-27632 also attenuates Htt toxicity in *Drosophila* and improves motor function in mice [10], [27]. Y-27632 blocks phosphorylation of profilin, an actin-binding protein that directly binds Htt, but not AR [28], [29]. Profilin strongly inhibits aggregation of ARN127 and Htt exon 1 in cells [29], and decreases polyglutamine-mediated toxicity in *Drosophila* [22]. The anti-aggregation effects of profilin depend on both its polyproline binding activity (required to bind Htt), and its ability to bind G-actin (required to suppress both Htt and AR aggregation) [29]. In this study, we have identified regions of ARN127 and Htt exon 1that bind filamentous actin (F-actin) *in vitro*, and investigate the effect of these regions on polyglutamine-dependent aggregation.

## Results

### ARN127 and Htt Exon 1 Bind F-Actin *In Vitro*

**Figure 1:**
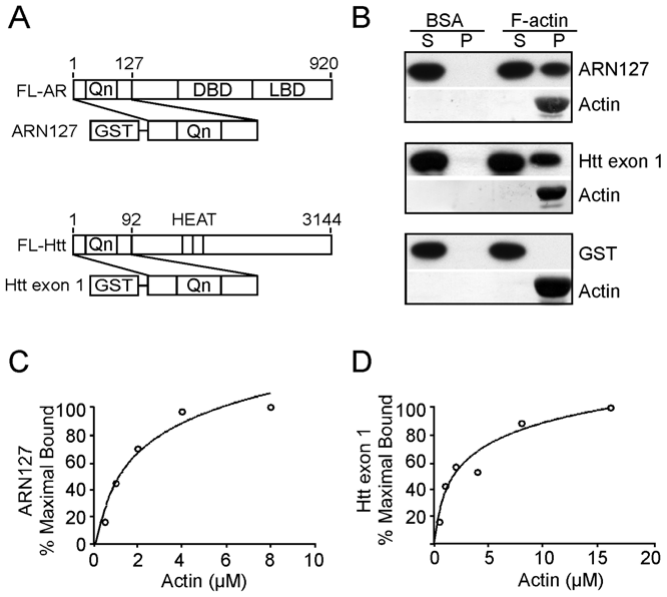
ARN127 and Htt exon 1 directly bind to F-actin *in vitro*. *A*, Schematic of GST-tagged N-terminal fragments of AR and Htt, GST-ARN127 and GST-Htt exon 1, in comparison to full-length AR (FL-AR) and full-length Htt (FL-Htt). *B*, GST-ARN127(25) and GST-Htt exon 1(25) co-sediment with F-actin *in vitro* but remain soluble in the absence of F-actin. 0.5 µM of precleared GST-ARN127(25) or .25 µM of precleared GST-Htt exon 1(25) was mixed with 4 µM of pre-polymerized F-actin. Mixtures were ultracentrifuged at 100,000 x g and supernatant and pellet fractions were analyzed via western blot (ARN127 and Htt exon 1) and Coomassie (actin). GST alone does not co-sediment with F-actin. *C*, Binding affinity of GST-ARN127(25) to F-actin. The F-actin co-sedimentation assay was performed with 0, .5 µM, 1 µM, 2 µM, 4 µM, and 8 µM F-actin and .016 µM GST-ARN127(25). Gels were quantified to determine the percent bound to F-actin. Percent maximal binding is reported. *D*, Binding affinity of GST-Htt exon 1(25) to F-actin. The F-actin co-sedimentation assay was performed with 0, .5 µM, 1 µM, 2 µM, 4 µM, 8 µM, and 16 µM F-actin and .15 µM GST-Htt exon 1(25). Gels were quantified to determine the percent bound to F-actin using Image J.

To test for a direct interaction between AR, Htt, and F-actin *in vitro*, we used an F-actin co-sedimentation assay with recombinant GST-ARN127 or GST-Htt exon 1 containing 25 glutamine repeats (Fig. 1A). Coomassie staining confirmed protein purity (Fig. S1A,E). Protein preparations were precleared by ultracentrifugation to remove any pre-existing aggregates. 0.5 µM GST-ARN127(25) or 0.25 µM GST-Htt exon 1(25) was incubated with F-actin (4 µM) that had been pre-polymerized *in vitro* for 1 hour at 25°C. As a control, proteins were incubated with an equal concentration of bovine serum albumin (BSA) instead of F-actin. After ultracentrifugation (100,000 x g), supernatant and pellet fractions were analyzed by western blot using antibody to GST. Western blot analysis was used rather than Coomassie stain because both proteins are very similar in size to G-actin (43 kDa). F-actin localized to the pellet fraction in all cases, as visualized by Coomassie stain (Fig. 1B). Both GST-ARN127(25) and GST-Htt exon 1(25) co-sedimented with F-actin while remaining soluble in its absence (Fig. 1B). GST alone did not co-sediment with F-actin (Fig. 1B). ARN127 also bound F-actin following cleavage of the GST-tag (Fig. S1B). We determined the binding affinity of GST-ARN127(25) and GST-Htt exon 1(25) for F-actin by using a constant amount of protein (16 nM for ARN127, 150 nM for Htt exon 1) mixed with increasing amounts of F-actin (Fig. 1C, D). GST-ARN127(25) bound to F-actin with an approximate dissociation constant (Kd) of 1 µM (Fig. 1C), while for GST-Htt exon1(25) the Kd was approximately 2 µM (Fig. 1D), comparable to other actin-binding proteins [30].

### Amino Acids 1-50 of ARN127 and 1-14 of Htt Exon 1 Mediate F-Actin Binding

**Figure 2:**
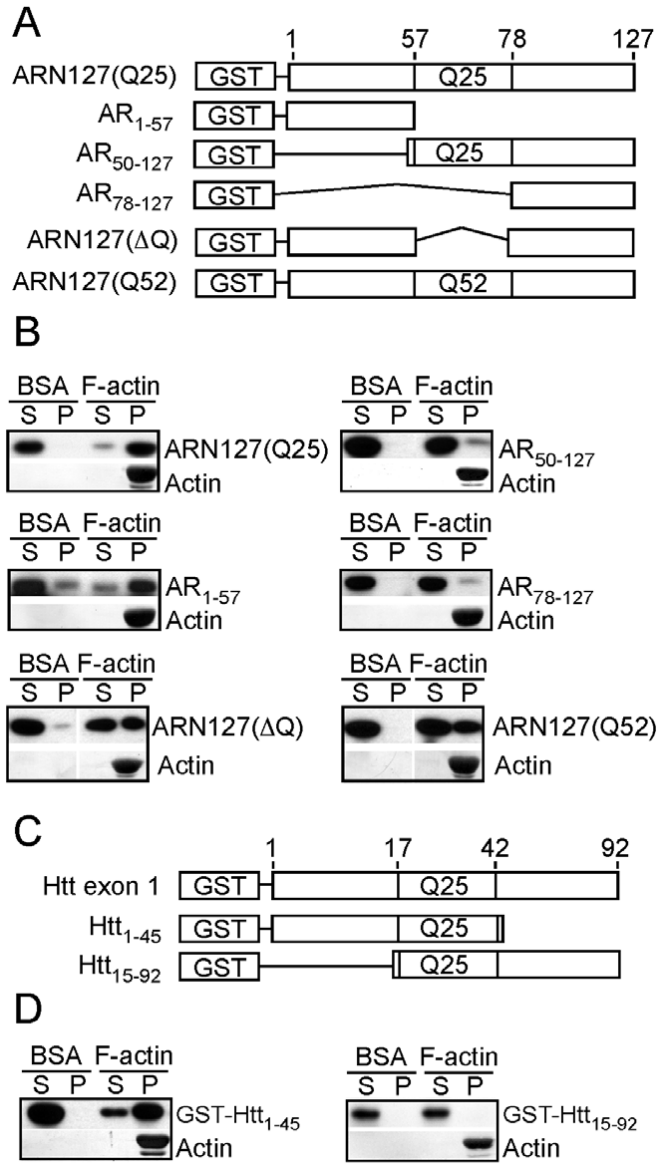
N50 of ARN127 and N14 of Htt exon 1 mediate F-actin binding *in vitro*. *A*, Schematic of GST-ARN127 and various truncation mutants. *B*, The N-terminus of ARN127 binds to F-actin *in vitro*. GST-tagged truncations of ARN127(25) (AR_1–57_, AR_50–127_, AR_78–127_ (20 nM)), were tested for binding to F-actin (4 µM). ARN127(25) and AR_1–57_ co-sediment with F-actin while AR_50–127_ and AR_78–127_ do not. GST-tagged ARN127(ΔQ) or ARN127(52) (0.5 µM) both co-sediment with F-actin. *C*, Schematic of GST-Htt exon 1 and GST-tagged truncation mutants. *D*, The N-terminus of Htt exon 1 binds to F-actin *in vitro*. GST-tagged Htt_1–45_ (0.1 µM) binds to F-actin (4 µM) while Htt_15–92_ (0.1 µM) does not.

We used deletion analysis to map the actin-binding regions of ARN127 and Htt exon 1 (Fig. 2A,C). Protein purity was assessed by Coomassie (Fig. S1A,C,E). Deletion of the polyglutamine domain of ARN127 and peptides C-terminal to the polyglutamine domain (AR_1–57_) had no appreciable effect on actin binding (Fig. 2B), while deleting the N-terminal peptides of AR (AR_50–127_, AR_78–127_) abolished binding (Fig. 2B). Constructs lacking the polyglutamine tract (ARN127(ΔQ)), or containing an expanded polyglutamine tract (ARN127(52)) bound actin equivalently (Fig. 2B). A very pure extract of AR_1–57_ cleaved from the GST-tag also bound F-actin efficiently (Fig. S1D). Deletion of peptides C-terminal to the polyglutamine tract of Htt exon 1 (Htt_1–45_) had no effect on binding while deletion of the first 14 amino acids (Htt_15–92_) abolished binding (Fig. 2C,D). Thus, the first 50 amino acids (N50) of AR and the first 14 amino acids (N14) of Htt mediate interactions with F-actin *in vitro*. There is no apparent homology between these regions.

### Sensitivity to Actin-Regulatory Pathways Requires the First 50 Amino Acids of ARN127

**Figure 3:**
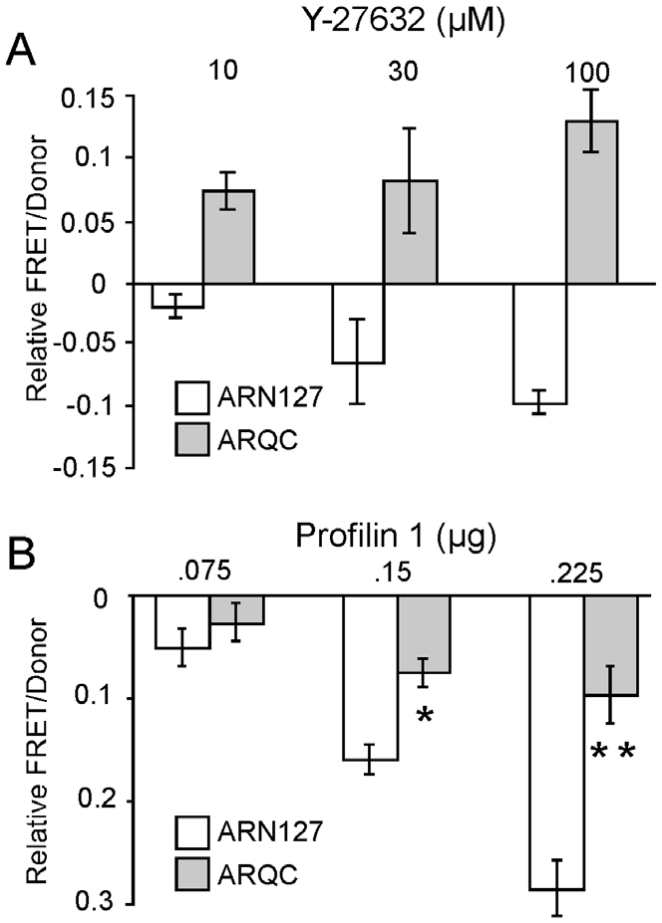
N50 of ARN127 mediates aggregation inhibition by actin- regulatory pathways. *A*, Y-27632 inhibits aggregation of ARN127(65)-CFP/YFP and increases ARQC(65)-CFP/YFP aggregation. Cells expressing either ARN127 or ARQC fused to CFP/YFP were treated with 10, 30, or 100 µM Y-27632 for 24 hours. Relative FRET/donor measurements represent the change in aggregation from compounds compared to untreated cells. Y-27632 suppressed ARN127(65)-CFP/YFP aggregation, whereas it increased ARQC(65)-CFP/YFP aggregation. *B*, Profilin 1 decreases aggregation of ARN127(65)-CFP/YFP dose-dependently. Profilin 1 was cotransfected with polyglutamine proteins at increasing amounts, .075, .15, or .225 µg. This response was diminished for ARQC(65)-CFP/YFP. (* = p<.01, ** = p<.0001, Student's t-test).

We have previously found that Y-27632, as well as a downstream ROCK target, profilin, inhibit ARN127 and Htt exon 1 aggregation [10], [25], [29]. Since both Y-27632 and profilin regulate actin assembly [31], [32], we tested whether deletion of the actin-binding region of ARN127 would alter its response to their inhibitory activities. We employed fluorescence resonance energy transfer (FRET) to quantify these effects [10]. We were only able to test AR using this assay, since deletion of the N14 region of Htt prevented significant FRET, possibly due to altered orientation of the CFP and YFP moieties. Expanded ARN127 and ARQC with 65 glutamines were tagged with fluorescence donor or acceptor tags, CFP or YFP, and co-transfected into HEK293 cells as previously described [10]. Y-27632 decreased ARN127(65)-CFP/YFP aggregation dose-dependently (Fig. 3A). Intriguingly, it increased the aggregation of ARQC(65)-CFP/YFP (Fig. 3A). Similarly, profilin 1 dose-dependently reduced ARN127(65)-CFP/YFP aggregation, but was much less effective on ARQC(65)-CFP/YFP aggregation (Fig. 3B). Thus, the N50 region of AR mediates effects of these actin regulators on polyglutamine-mediated aggregation.

### N50 of ARN127 Affects Inclusion Type, Number, and Distribution

**Figure 4:**
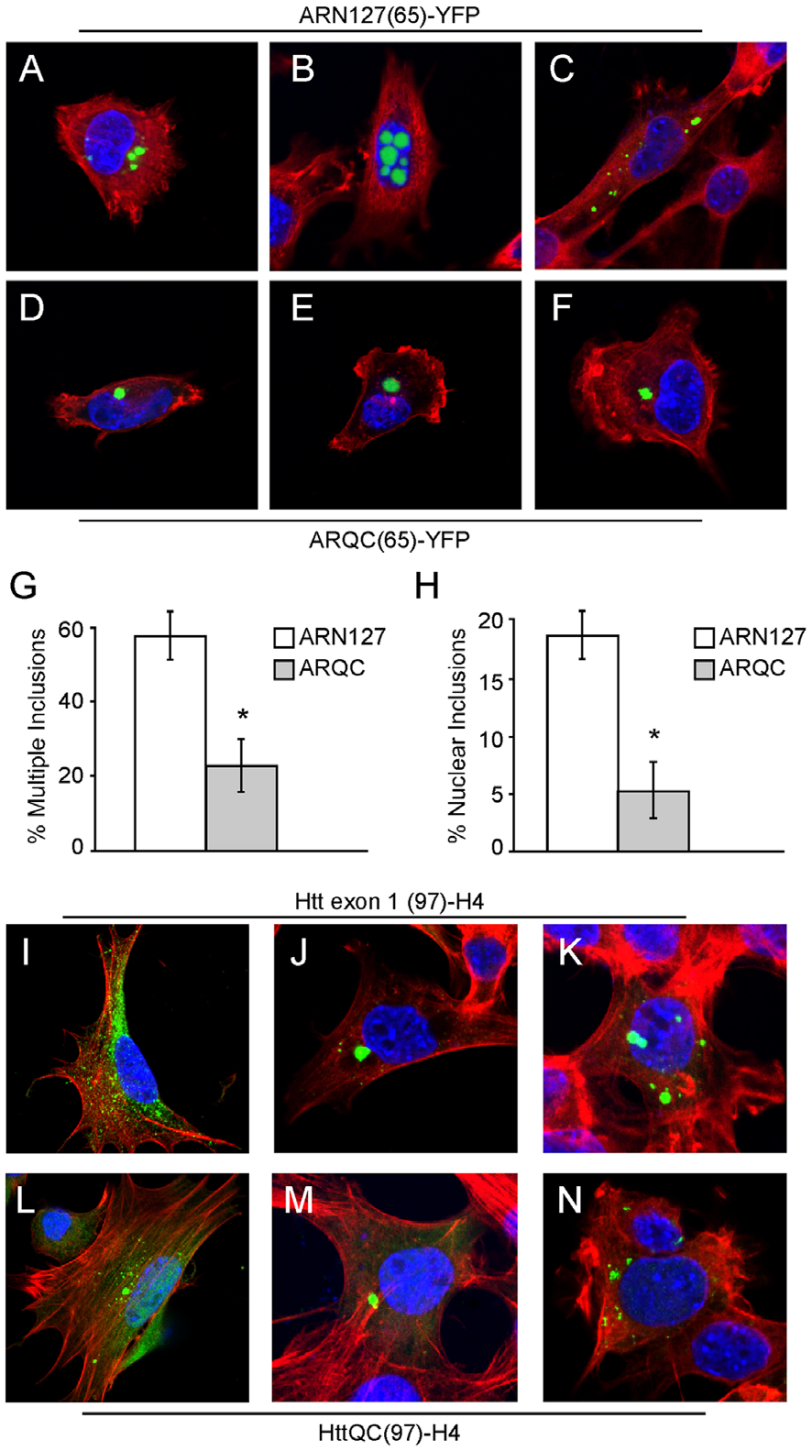
N50 of ARN127 influences inclusion type, number, and distribution. *A–C*, Confocal images of ARN127(65)-YFP inclusions in C17.2 cells at 48 h (60X). *D–F*, Confocal images of ARQC(65)-YFP inclusions in C17.2 cells at 48 h (60X). YFP-tagged proteins are in green, F-actin is stained with rhodamine-phalloidin (red) and DNA is stained with DAPI (blue). *G*, ARN127(65)-YFP more often forms multiple inclusions per cell than ARQC(65)-YFP (* = p<.002, Student's t-test). *H*, ARN127(Q65)-YFP more often forms nuclear inclusions per cell than ARQC(65)-YFP (* = p<.002, Student's t-test). Averages are from three separate transfections, counting at least 100 cells each. *I-K*, Confocal images of Htt exon 1(97)-H4 in C17.2 cells at 48 h (60X). *L-N*, Confocal images of HttQC(97)-H4 in C17.2 cells at 48 h (60X). Immunofluorescence of HA-tagged Htt is in green, F-actin is stained with rhodamine-phalloidin (red) and DNA is stained with DAPI (blue). We did not observe significant differences in patterns of inclusion formation between the two Htt constructs.

**Figure 5:**
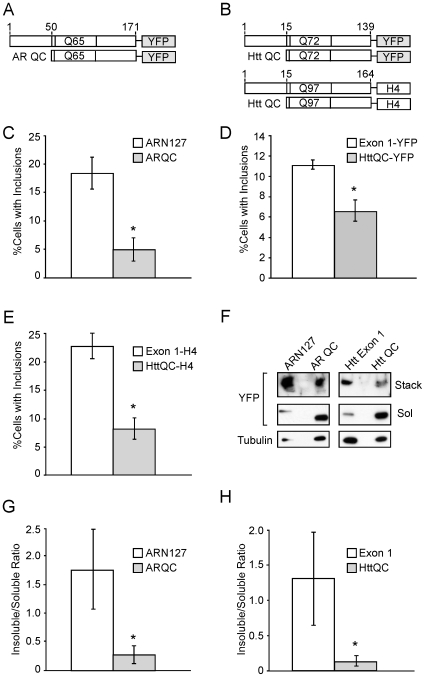
N50 of ARN127 and N14 of Htt exon 1 increase inclusion formation and detergent insolubility of aggregates. HEK293 cells were transfected with the indicated constructs and evaluated after 24 h. *A*, Schematic of AR fusion proteins, not to scale. *B*, Schematic of Htt fusion proteins, not to scale. The H4 sequence represents HIS-HA-HA-HIS epitopes. *C*, ARN127(65)-YFP forms more inclusions than ARQC(65)-YFP (* = p<.0025, Student's t-test). *D*, Htt exon 1(72)-YFP forms more inclusions than HttQC(72)-YFP (* = p<.0025, Student's t-test). *E*, Htt exon 1(97)-H4 forms more inclusions than HttQC(97)-H4 (* = p<.005, Student's t-test). *F*, ARN127(65)-YFP and Htt exon 1(72)-YFP form more SDS-insoluble aggregates than ARQC(65)-YFP and HttQC(72)-YFP. HEK293 cells were transiently transfected with the indicated constructs. After 24 h, cells were lysed in 2% SDS sample buffer, and subjected to SDS-PAGE and western blot with YFP antibody. Stack indicates the SDS-insoluble higher molecular weight aggregates trapped in the stacking gel; Sol indicates the SDS-soluble monomers. Tubulin indicates loading control. Deletion of amino terminal peptides reduced the overall proportion of SDS-insoluble material detected in the stacking gel. *G*, Quantification of relative insoluble to soluble fractions of ARN127 vs. ARQC (n = 3, * = p<.05, Student's t-test). *H*, Quantification of relative insoluble to soluble fractions of Htt exon 1 vs. HttQC (n = 3, * = p<.05, Student's t-test). Quantification by Image J.

To better characterize the behavior of expanded AR and Htt peptides within the cell, and to determine the influence of the actin-binding regions, we transfected expanded forms of each construct into C17.2 neural precursor cells, which were used for their ease of imaging. Identical results were obtained with HEK293 cells (data not shown). We observed clear differences between inclusions formed by ARN127(65)-YFP vs. ARQC(65)YFP. ARN127(65)-YFP formed many different types of inclusions (single, multiple, nuclear, cytoplasmic), whereas ARQC(65)YFP tended to form one perinuclear inclusion (Fig. 4A–F). After 48 h post-transfection, ARN127(65)-YFP expression resulted in multiple inclusions per cell in ∼57% of cells vs. ∼23% for ARQC(65)-YFP (Fig. 4G). ARN127(65)-YFP produced nuclear inclusions in ∼19% of cells vs. ∼5% of cells for ARQC(65)-YFP (Fig. 4H). These data are consistent with the idea that protein interactions mediated by the N50 domain of AR could alter the type of protein aggregates, as well as their subcellular localization. In contrast, we did not observe obvious inclusion differences between Htt exon 1 and HttQC. Htt exon 1(72)-YFP and HttQC(72)-YFP formed inclusions with similar visual characteristics (data not shown) as did Htt exon 1(97)-H4 and HttQC(97)-H4 (Fig. 4I-N), which contain a HIS-HA-HA-HIS epitope tag [33] (Fig. 5B).

### N50 of ARN127 and N14 of Htt Exon 1 Promote Inclusion Formation

To quantify the effect of N50 of ARN127 and N14 of Htt exon 1 on intracellular aggregation, we transiently transfected HEK293 cells with expanded AR constructs (ARN127(65)-YFP or ARQC(65)-YFP) or Htt constructs (Htt exon 1(72)-YFP or HttQC(72)-YFP; Htt exon 1(97)-H4 or HttQC(97)-H4, which contain a HIS-HA-HA-HIS epitope tag) and cultured the cells for 24 h (Fig. 5A–E). In each case, the QC proteins produced significantly fewer visible inclusions (Fig. 5C–E). Biochemical analysis of total cell lysates after 24 h in 2% SDS revealed that the soluble fractions of both ARQC(65)-YFP and HttQC(72)-YFP were enriched, while the SDS-insoluble aggregates, were depleted (Fig. 5F–H). Thus, N50 of ARN127 and N14 of Htt exon 1 each promote the formation of inclusions and SDS-insoluble aggregates.

### N50 of ARN127 and N14 of Htt Exon 1 Promote SDS-Insoluble Aggregates

**Figure 6:**
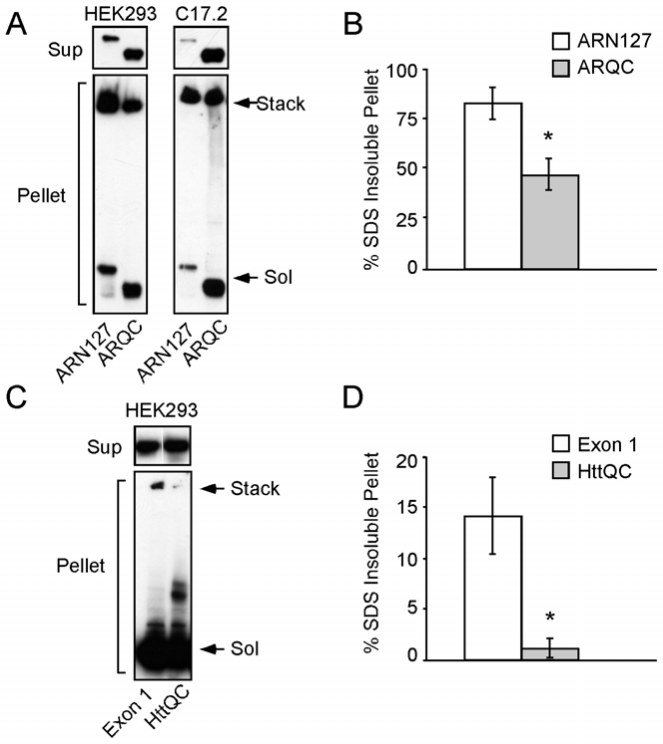
N50 of ARN127 and N14 of Htt exon 1 increase SDS-insoluble aggregate formation. *A*, ARN127(65)-YFP forms more SDS-insoluble aggregates than ARQC(65)-YFP. HEK293 and C17.2 cells were transiently transfected with either ARN127(65)-YFP or ARQC(65)-YFP. After 24 h, cells were syringe lysed and fractionated by centrifugation at 15,000 x g. Supernatant and pellet fractions were then resuspended in 2% SDS sample buffer and subjected to SDS-PAGE and western blot with YFP antibody. The soluble portions are shown in the upper panels, while the insoluble portions are shown in the lower panels. Stack indicates the SDS-insoluble higher molecular weight aggregates in the stacking gel; Sol indicates the SDS-soluble monomers. *B*, Quantification of SDS-insoluble aggregates for ARN127(65)-YFP and ARQC(65)-YFP from HEK293 cells after 24 h. The insoluble fraction of ARN127(65)-YFP has significantly more SDS-insoluble material than ARQC(65)-YFP (n = 3, * = p<.001, Student's t-test). *C*, Htt exon1(97)-H4 forms more insoluble aggregates than HttQC(97)-H4 in HEK293 cells after 24 h. Cells were treated as above and subjected to SDS-PAGE and western blot with HA antibody. *D*, Quantification of SDS-insoluble aggregates for Htt exon1(97)-H4 and HttQC(97)-H4 from HEK293 cells after 24 h. The insoluble fraction of Htt exon 1(97)-H4 has significantly more SDS-insoluble material than HttQC(97)-H4 (n = 3, * = p<.005, Student's t-test).

The distinct effects of actin-binding regions on AR and Htt inclusion formation suggested that they might also change aggregate characteristics. Thus, we transfected HEK293 cells with the various constructs as above. 24 h post-transfection the cells were disrupted via syringe lysis and fractionated by centrifugation (15,000 x g) to compare soluble vs. insoluble species. Supernatant and pellet fractions were resuspended in 2% SDS and resolved by SDS-PAGE and western blot. All protein in the supernatant fraction was completely solubilized by SDS (Fig. 6A). In contrast, both ARN127(65)-YFP and ARQC(65)-YFP peptides that partitioned into the insoluble pellet contained mixtures of SDS-soluble and insoluble proteins (Fig. 6A,B). The aggregates of ARQC(65)-YFP were much more readily dissociated in 2% SDS compared to ARN127(65)-YFP (Fig. 6A,B). Parallel experiments in C17.2 cells revealed similar results (Fig. 6A). We observed similar phenomena for Htt exon 1(97)-H4 vs. HttQC(97)-H4 (Fig. 6C). The pellet fraction containing HttQC(97)-H4 was significantly more SDS-soluble (Fig. 6C,D). Thus flanking sequences of AR and Htt influence both the propensity for protein misfolding and the biochemical characteristics of the aggregates that result.

## Discussion

In this study, we have focused on aggregation-prone fragments of AR (ARN127) and Htt (Htt exon 1), identifying amino acids 1–50 (N50) of ARN127 and 1–14 (N14) of Htt exon1 as regions that influence polyglutamine-dependent aggregation. These regions were originally identified because they mediate binding to F-actin *in vitro*. However, we did not observe co-localization of ARN127 or Htt exon 1 with the F-actin cytoskeleton (data not shown). This could reflect lack of significant binding, or that intracellular binding to F-actin is transient. Deletion of the N50 region of ARN127 or the N14 region of Htt decreased the total number of inclusions formed, and the aggregates that did form were more SDS-soluble. Further, the N50 region of AR regulates the characteristics of inclusions formed and mediates its responsiveness to the anti-aggregation effects of Y-27632 and profilin. Thus, the flanking sequence of the polyglutamine region does not simply alter the propensity to form a polyglutamine aggregate, but can directly affect its aggregation rate and responses, the subcellular localization of the inclusions, and the biochemical characteristics of the aggregates.

The importance of flanking sequences on Htt exon 1 has previously been demonstrated: flanking sequences have been shown to directly regulate aggregation, toxicity, and the morphology of inclusions [13], [34], [35]. Given the importance of primary amino acid sequence on polyglutamine aggregation *in vitro* [12], [14], we cannot exclude that the intracellular aggregation differences observed are solely due to altered intrinsic aggregation kinetics. However, this cannot explain all observable differences noted, such as altered distribution of polyglutamine inclusions. We have found that peptides containing either the first 50 amino acids of AR or the first 14 amino acids of Htt partition to Triton-insoluble cell fractions independent of their inherent solubility (Figs. S2, S3), suggesting that these regions could mediate protein interactions with macromolecular structures in the cytoplasm. Indeed, the first 17 amino acids of Htt have previously been implicated as a pro-aggregation-domain, a cytoplasm-targeting domain, and have been shown to bind mitochondria, the endoplasmic reticulum, and the Golgi apparatus [35], [36]. This region is highly conserved across diverse species. Taken together, these studies imply that this region is likely to mediate protein interactions that play an important role in determining aggregation properties of the Htt peptide.

Although we did not detect binding of AR or Htt to F-actin in cells, the *in vitro* binding of the Htt N-terminus to F-actin is intriguing in light of Htt's capacity also to bind the actin remodeling factor profilin. Profilin/Htt interaction is most likely mediated by Htt's polyproline domain, immediately distal to the polyglutamine region in the Htt peptide [28], [29]. Analysis of the first 17 amino acids of Htt reveal a striking similarity to another actin-binding peptide, Lifeact [37]. These observations may indicate a normal, perhaps transient, function for Htt in the regulation of the actin cytoskeleton, although this remains to be tested.

In this study, we find that sequences independent of the polyglutamine tract have profound effects on subcellular localization, detergent solubility, and inclusion formation of polyglutamine peptides. Given the apparent importance of these regions for protein interaction, this implies that modifiers of protein aggregation are likely to be found within the set of AR and Htt interacting proteins. Indeed, the recent finding that Htt-interacting proteins are enriched for genetic modifiers of toxicity is consistent with this idea [38]. Further study of AR and Htt binding proteins thus may reveal mechanisms that specify particular aggregation pathways and pathogenic features of each disease.

## Materials and Methods

### Constructs

Bacterial expression vectors for ARN127 Htt exon 1 and various derivatives were constructed via PCR amplification and subcloned in the pGEX4T1 backbone (Amersham Biosciences). Similarly, for mammalian expression vectors, ARQC and HttQC plasmids were constructed via PCR amplification and subcloned into pECFP-N1, pEYFP-N1 (Clontech), or pcDNA.3 backbones. GST-Htt exon 1 constructs were originally obtained from Paul Muchowski and mammalian pcDNA3-Htt-H4 constructs were originally obtained from Joan Steffan.

### Cell Culture and Transfection

HEK293 cells were plated at 250,000 cells per well in a 24-well dish and transfected with .3 µg total DNA with Lipofectamine and Plus reagent (Invitrogen) according to the manufacturer's instructions. C-17.2 cells were plated at 100,000 cells per 24-well and transfected with .6 µg DNA with Lipofectamine 2000 (Invitrogen) according to the manufacturers instructions. Cells were harvested at indicated times.

### FRET

All FRET measurements were carried out 48 hours after transfection of HEK293 cells, read in 96-well cell-culture plates by a fluorescence plate reader (Tecan). HEK293 cells were transfected with .075 µg total ARN127CFP/YFP DNA in a 1∶3 donor:acceptor ratio. Profilin DNA was co-transfected with AR constructs at concentrations of .075 µg, .15 µg, or .225 µg. pcDNA3 backbone vector was also co-transfected for a constant final concentration of .3 µg. Y-27632 was added at the indicated concentration for 24 h prior to FRET measurements[29].

### Confocal Microscopy

All images were acquired on a C1sl confocal microscope (Nikon Instruments Inc.).

### Immunofluorescence

C17.2 cells were mounted on polyornithine-coated glass coverslips 48 hours after transfection at a density of 20,000 cells per coverslip for 60X imaging. Cells were fixed in 4% paraformaldehye, treated with .5% Triton, and blocked in 5% BSA for 1 hour. Coverslips were treated with HA antibody (1∶500, Covance) overnight at 4°C, rinsed 4X and washed 3X with %1 TBS-Tween, and treated with donkey-anti-mouse Alexa-Fluor 488 (1∶400, Molecular probes) for 1 h at 37°C. Coverslips were rinsed 4X and washed 2X with %1 TBS-Tween. F-actin was visualized with rhodamine-conjugated phalloidin (1∶300, Molecular Probes) and the nucleus was stained with DAPI (Sigma). Coverslips were mounted with anti-fade mounting media (Invitrogen) and analyzed 24 h later.

### Inclusion Counting

At least 100 HEK293 cells per transfection (3) were examined for total number of inclusions formed after 24 h. Approximately 100 C17.2 cells per transfection (3) were counted for inclusions. Cells were identified as having nuclear, cytoplasmic, or multiple inclusions, or a combination.

### Protein Purification

GST-ARN127 plasmids were grown in *E. Coli* Rosetta 2 (DE3) competent cells (Novagen). Protein expression was induced with 1 mM (isopropyl β-d-thiogalactoside) IPTG for 3 h at 37°C. GST-Htt plasmids were grown in *E. Coli* SURE competent cells (Stratagene). Protein expression was induced with 1 mM IPTG for 3H at 30°C. Bacterial pellets were resuspended in resuspension buffer (PBS, .05% Tween, 1 mM PMSF, protease inhibitor tablet (Roche)) and lysed by sonication and 1% Triton. GST-tagged proteins were precipitated with glutathione sepharose (Amersham Biosciences) and eluted with equal volumes of elution buffer (50 mM Tris-HCl pH. 8 m 10 mM reduced glutathione). For cleavage of the GST-tag, 37.5 units of thrombin protease (Amersham Biosciences) was used for .5 ml of GST-bound glutathione sepharose 4B (Amersham Sepharose) at 4°C overnight. Thrombin was removed from cleaved AR with benzamidine sepharose 6B (Amersham Biosciences). Protein concentration was quantified via Bradford assay and Coomassie staining via Image J.

### F-Actin Co-Sedimentation

Non-muscle human actin (Cytoskeleton) was polymerized according to the manufacturer's instructions. 10 mg/ml of G-actin was polymerized with polymerization buffer (10X∶ 500 mM KCl, 20 mM MgCl2, 10 mM ATP) in general actin buffer (5 mM Tris-HCl pH8.2 mM CaCl2, .5 mM DTT, .2 mM ATP) for 1 h at RT. Equal molar ratios of unlabeled phalloidin (Molecular Probes) were added to stabilize filaments. Recombinant, purified GST-tagged proteins were precleared via ultracentrifugation (100,000 x g for 30 min at 4°C). Proteins were added to a pre-polymerized F-actin (4 uM) or a BSA control for 1 h on ice. Mixtures were ultracentrifuged for 30 min at 100,000 x g at 4°C. Supernatants and pellets were subjected to SDS-PAGE. F-actin pellets were visualized via Coomassie stain. GST-proteins were probed via western blot using GST antibody (Santa Cruz Bioscience).

### Detergent Fractionation

For unexpanded AR and Htt constructs, HEK293 cells were harvested 24 h post-transfection. Cell pellets were lysed in 130 µl cold lysis buffer (PBS, 1 % Triton, 5 mM EDTA, protease inhibitor cocktail (Roche)) and subjected to high-speed ultracentrifugation (100,000 x g). For expanded AR and Htt constructs, HEK293 cells were harvested 24 h later post-transfection. Cell pellets were lysed by syringe passage in cold resuspension buffer (PBS, 5 mM EDTA, protease inhibitor cocktail (Roche)) and subjected to table-top centrifugation (15,000 x g). Supernatant and pellets fractions were denatured in 2% SDS buffer, 25 mM DTT, and boiled for 10 min. For crude cell-lysates, cell pellets were directly lysed in 200 µl of 2% SDS, 25 mM DTT and boiled for 10 minutes. All lysates were subjected to SDS-PAGE and probed via western blot using GFP antibody (Santa Cruz Biotechnology) or HA antibody (Covance). Supernatants, pellets and higher molecular weight bands were quantified using Image J.

### Antibodies

Anti-rabbit N-20 antibody (1∶1000, Santa Cruz) was used for the detection of cleaved AR products. Anti-rabbit GFP-antibody (Santa Cruz) or anti-mouse HA-antibody (Covance) was used for the detection of YFP-tagged or HA-tagged proteins at 1∶2000 dilution for soluble proteins or 1∶1000 dilution for insoluble higher-molecular weights. Anti-mouse MW7 was used to detect the C-terminus of Htt at 1∶2000 dilution for soluble proteins or 1∶1000 dilution for insoluble higher-molecular weights.

Anti-mouse GST-antibody (1∶2000, Santa Cruz) was used for the detection of GST-tagged proteins.

### In Vitro Solubility

GST-ARN127(25), GST-ARNQ(32), GST-ARQC(36), GST-Htt exon 1(25), and GST-HttQC(25) were recombinantly purified and quantified as described above. 1.25 mg/ml of GST-AR or .75 mg/ml of GST-Htt purified proteins were ultracentrifuged (100,000 x g) and incubated at 37°C for 1 h to promote misfolding. For cleavage of the GST tag, 1.0 mg/ml of precleared GST-AR peptide were incubated with 1 NIH unit of thrombin (Invitrogen) overnight at 4°C. Proteins were ultracentrifuged and supernatant and pellet fractions were resuspended in SDS sample buffer and subjected to SDS-PAGE. Proteins were analyzed via Coomassie stain.

## Supporting Information

Figure S1 Purity of Recombinantly Purified Proteins. A, Coomassie stain of GST-ARN127(25), GST-ARN127(ΔQ), and GST-ARN127(52). Lower molecular weight (LMW) contaminants are indicated B, Cleaved ARN127(25) co-sediments with F-actin but is soluble in the absence of F-actin. GST-tagged ARN127(25) was treated with thrombin to cleave off the GST tag. 1 µM of thrombin-cleaved ARN127(25) was mixed with 5 µM F-actin as in Figure 1. Western blot was used to detect the N-terminus of AR (N-20 antibody) or Coomassie for actin. C, Coomassie stain of GST and GST-AR fragments, AR1-57, AR50-127, and AR78-127, D, Cleaved AR1-57 cosediments with F-actin but is soluble in the absence of F-actin. GST-tagged AR1-57 was treated with thrombin to cleave off the GST tag. 1 µM of thrombin-cleaved AR1-57 was mixed with 5 µM F-actin. Western blot was used to detect the N-terminus of AR (N-20 antibody) or Coomassie stain for actin. E, Coomassie stain of GST-Htt exon 1(25), GST- Htt1-45, and GST- Htt15-92.
(5.47 MB TIF)

Figure S2 N50 of ARN127 and N14 of Htt exon 1 mediate macromolecular interactions. A, Schematic of AR and Htt peptides fused to YFP. B, The N-terminus of AR mediates macromolecular interactions. HEK293 cells were transfected with YFP fusion proteins, lysed in 1% Triton, and subjected to ultracentrifugation (100,000 x g). Supernatant and pellet fractions were analyzed via western blot with YFP antibody. ARN127(25)-YFP (top band) and ARNQ(25)-YFP are present in the Triton-insoluble pellets of HEK293 cells, while ARQC(25)-YFP is not. Tubulin indicates loading control. C, Deletion of the N14 region of Htt exon 1(25) does not affect Triton solubility. Supernatant and pellet fractions were analyzed via western blot with MW7 antibody (detects the C-terminus of Htt). Tubulin indicates loading control. D, The first 17aa of Htt alone fused to YFP cause it to become insoluble in cell lysates. Blots are probed with YFP. Tubulin indicates loading control.
(9.60 MB TIF)

Figure S3 Inherent Solubility of GST peptides. A, There are no differences in the inherent solubility of AR peptides. GST-tagged ARN127(25), ARNQ(32), and ARQC(36) are present in equal amounts in the pellet fractions after ultracentrifugation. B, Cleavage of AR peptides from GST does not unmask drastic differences in inherent solubility. GST was cleaved from ARN127(25), ARNQ(32), and ARQC(36) with thrombin protease at 4°C overnight. Peptides were ultracentrifuged (100,000 x g) and supernatant and pellet fractions were analyzed via SDS-PAGE and Coomassie stain.
(8.09 MB TIF)
