# Supplementary figures and images for: F-Actin Binding Regions on the Androgen Receptor and Huntingtin Increase Aggregation and Alter Aggregate Characteristics
Source: PLoS One. 2010 Feb 4;5(2):e9053. doi: 10.1371/journal.pone.0009053 (PMC2816219; doi:10.1371/journal.pone.0009053)

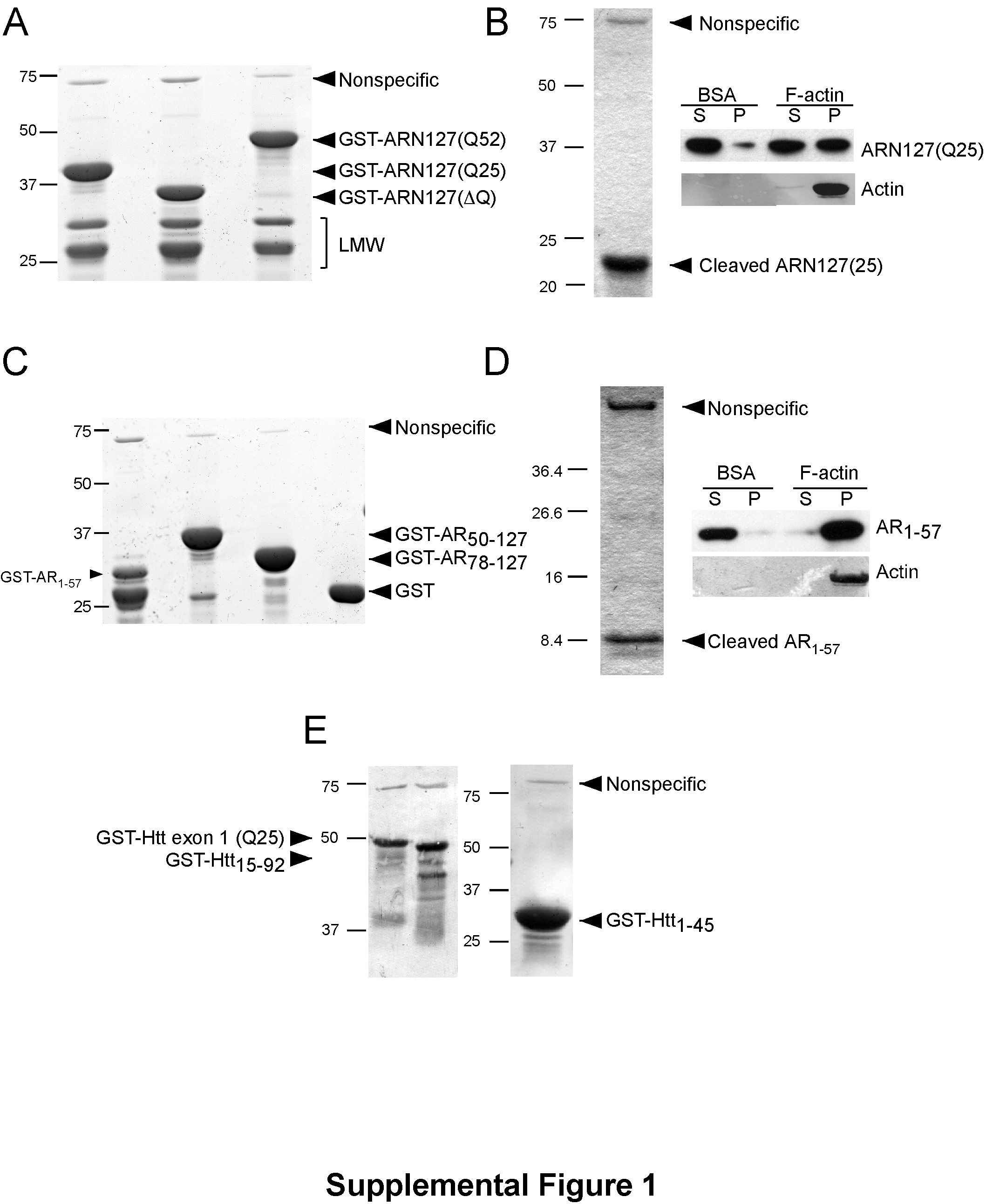

Supplement: Figure S1 — Purity of Recombinantly Purified Proteins. A, Coomassie stain of GST-ARN127(25), GST-ARN127(ΔQ), and GST-ARN127(52). Lower molecular weight (LMW) contaminants are indicated B, Cleaved ARN127(25) co-sediments with F-actin but is soluble in the absence of F-actin. GST-tagged ARN127(25) was treated with thrombin to cleave off the GST tag. 1 µM of thrombin-cleaved ARN127(25) was mixed with 5 µM F-actin as in Figure 1. Western blot was used to detect the N-terminus of AR (N-20 antibody) or Coomassie for actin. C, Coomassie stain of GST and GST-AR fragments, AR1-57, AR50-127, and AR78-127, D, Cleaved AR1-57 cosediments with F-actin but is soluble in the absence of F-actin. GST-tagged AR1-57 was treated with thrombin to cleave off the GST tag. 1 µM of thrombin-cleaved AR1-57 was mixed with 5 µM F-actin. Western blot was used to detect the N-terminus of AR (N-20 antibody) or Coomassie stain for actin. E, Coomassie stain of GST-Htt exon 1(25), GST- Htt1-45, and GST- Htt15-92. (5.47 MB TIF) [file pone.0009053.s001.tif]

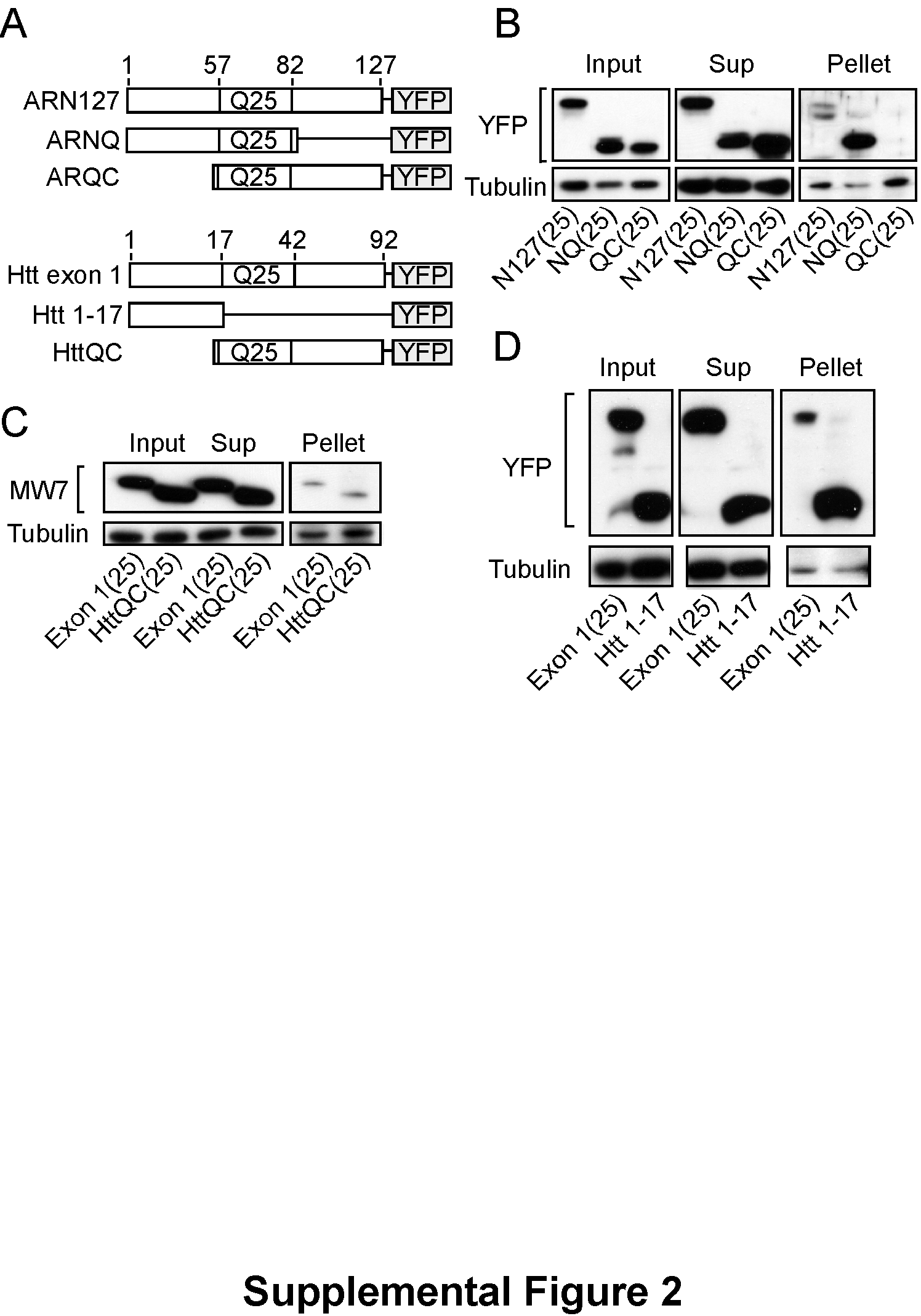

Supplement: Figure S2 — N50 of ARN127 and N14 of Htt exon 1 mediate macromolecular interactions. A, Schematic of AR and Htt peptides fused to YFP. B, The N-terminus of AR mediates macromolecular interactions. HEK293 cells were transfected with YFP fusion proteins, lysed in 1% Triton, and subjected to ultracentrifugation (100,000 x g). Supernatant and pellet fractions were analyzed via western blot with YFP antibody. ARN127(25)-YFP (top band) and ARNQ(25)-YFP are present in the Triton-insoluble pellets of HEK293 cells, while ARQC(25)-YFP is not. Tubulin indicates loading control. C, Deletion of the N14 region of Htt exon 1(25) does not affect Triton solubility. Supernatant and pellet fractions were analyzed via western blot with MW7 antibody (detects the C-terminus of Htt). Tubulin indicates loading control. D, The first 17aa of Htt alone fused to YFP cause it to become insoluble in cell lysates. Blots are probed with YFP. Tubulin indicates loading control. (9.60 MB TIF) [file pone.0009053.s002.tif]

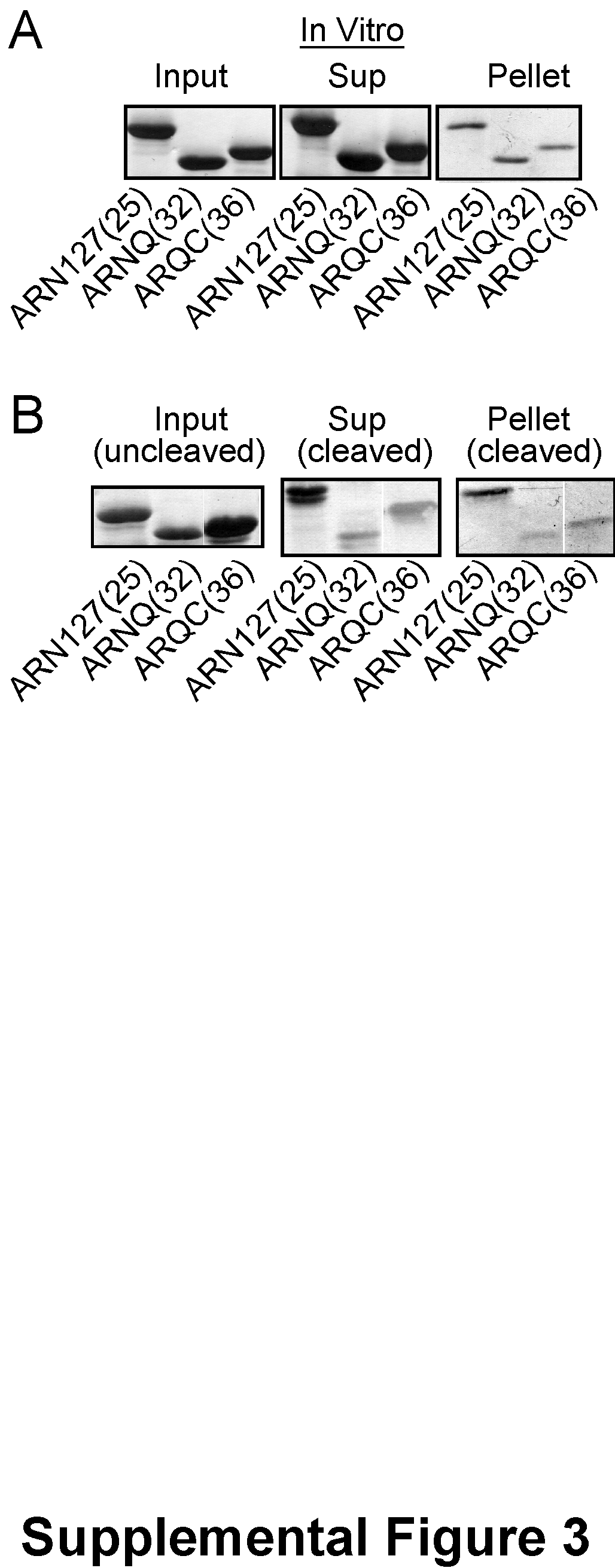

Supplement: Figure S3 — Inherent Solubility of GST peptides. A, There are no differences in the inherent solubility of AR peptides. GST-tagged ARN127(25), ARNQ(32), and ARQC(36) are present in equal amounts in the pellet fractions after ultracentrifugation. B, Cleavage of AR peptides from GST does not unmask drastic differences in inherent solubility. GST was cleaved from ARN127(25), ARNQ(32), and ARQC(36) with thrombin protease at 4°C overnight. Peptides were ultracentrifuged (100,000 x g) and supernatant and pellet fractions were analyzed via SDS-PAGE and Coomassie stain. (8.09 MB TIF) [file pone.0009053.s003.tif]
